# The Effect of Clothing on Vitamin D Status, Bone Turnover Markers, and Bone Mineral Density in Young Kuwaiti Females

**DOI:** 10.1155/2019/6794837

**Published:** 2019-06-23

**Authors:** Fatima Ibrahim Al-Yatama, Fatemah AlOtaibi, Maie Dawoud Al-Bader, Kamal A. Al-Shoumer

**Affiliations:** ^1^Department of Medical Laboratories, Faculty of Allied Health, Kuwait University, Kuwait; ^2^Department of Physiology, Faculty of Medicine, Kuwait University, Kuwait; ^3^Department of Medicine, Faculty of Medicine, Kuwait University, Kuwait

## Abstract

Many Arab women in the Gulf region cover their bodies for cultural and religious reasons, limiting the skin's exposure to sunlight and therefore its ability to synthesize vitamin D. The aim of this study is to determine whether the clothing style of Kuwaiti premenopausal women affects their vitamin D status, bone marker expression, and bone density. Three groups of healthy unmarried single Kuwaiti females (20-35 years old; n=30 per group) were recruited randomly from the general community: a control group who wear Western-style clothing (unveiled group), a group who wear a hejab that covers the whole body except for the face and hands (hejab group), and a group who wear a black veil with the entire body covered (veiled group). Bone mineral density (BMD), bone markers (procollagen type 1 N-terminal propeptide [P1NP], osteocalcin, and *β*-CrossLaps), 25-hydroxy vitamin D, intact parathyroid hormone [iPTH], and calcitonin were measured. The bone marker osteocalcin was significantly higher in the hejab group compared to the control group, whereas P1NP and *β*-CrossLaps were significantly higher in the veiled group compared to the control group. 25-hydroxy vitamin D, iPTH, calcitonin, and BMD were not significantly different across the three groups despite the observed elevation in bone turnover markers. The majority of participants in all three groups exhibited vitamin D deficiency; however, the lowest vitamin D levels were observed among the hejab and veiled participants. These findings suggest that clothing style may contribute to vitamin D deficiency in young Kuwaiti women.

## 1. Introduction

Vitamin D is a steroid hormone that affects human health [1]. Vitamin D can be acquired from the diet, e.g., in fish oil or fortified dairy products, but approximately 90% of the body's vitamin D is synthesized via skin after exposure to solar ultraviolet B (UVB) radiation. Exposure of the skin to UVB radiation is therefore important for attaining adequate vitamin D levels. Skin pigmentation has been reported to affect synthesis of vitamin D such that darker skin requires longer exposure to sunlight than fairer skin [2]. Age and clothing style also have been reported to affect vitamin D synthesis [3, 4].

Vitamin D is involved in bone metabolism [5] and is positively correlated with bone mineral density (BMD).

Vitamin D status is assessed by measuring vitamin D levels in plasma or serum. Although there has been much debate over the definition of adequate and optimal vitamin D status based on blood vitamin D levels, there is general consensus that values < 25 nmol/l (10 ng/ml) indicate deficiency and ≥50 nmol/l (20 ng/ml) indicate sufficiency [2].

Vitamin D deficiency has been associated with several bone-related diseases, including rickets, osteomalacia, and osteoporosis [6, 7]. Vitamin D deficiency has been linked to reproductive disorders, including polycystic ovary syndrome, uterine leiomyomas, and endometriosis [8]. Vitamin D deficiency also has been linked to colorectal cancer [9], diabetic peripheral neuropathy, and retinopathy [10, 11], susceptibility to multiple sclerosis, increased severity of respiratory distress syndrome, infant bronchopulmonary dysplasia [12], and mental disorders [13]

Vitamin D deficiency is pandemic, occurring in individuals of all ages and ethnicities [14]. Vitamin D deficiency has been observed even in countries with adequate sunshine, including Asia, North Africa, and the Middle East [15, 16].

It has been suggested that the high prevalence of vitamin D deficiency in Arab women, especially those residing in the Gulf region, is due to low exposure to sunlight. More than 95% of Arab women cover their bodies for cultural and/or religious reasons. The clothing style of Arab women may therefore cause low vitamin D levels, leading to vitamin D deficiency [17]. The present study focused on the effect of clothing style on vitamin D levels among young healthy Kuwaiti females. This study also assessed the impact of clothing style on BMD, which has not been previously assessed.

## 2. Materials and Methods

### 2.1. Participants

This study recruited healthy premenopausal single Kuwaiti females between the ages of 20 and 35 years, with skin colour type IV (olive skin). Written informed consent was obtained from each subject and the study was approved by the Ethical Committee of the Faculty of Medicine, Health Science Center and the Ministry of Health. The study was conducted in accordance with the declaration of Helsinki. All participants completed a questionnaire that included questions about their age; sex; average duration of daily indoor and outdoor sunlight exposure; past medical history, including family history of vitamin D deficiency; dietary habits; and clothing style. Participants were excluded for hepatic or renal disease, metabolic bone disease, malabsorption, oligomenorrhea, type I diabetes, hypercortisolism, malignancy, >1 week of immobility, or medications known to influence bone metabolism. Participants were classified into three groups (n=30 per group) on the basis of their clothing style. Group 1 included female participants who reported wearing Western-style clothing (control unveiled group). Group 2 included female participants who reported that they have worn the hejab since puberty with their face and hands exposed (hejab group). Group 3 included female participants who reported that they have worn the black veil since puberty with the majority of their body covered (veiled group).

### 2.2. Sample Collection

Fasting plasma and serum samples were collected from each participant and aliquoted and stored at -20°C for subsequent analysis.

### 2.3. Bone Mineral Densitometry

Bone density was measured in the lumbar spine (L2-L4), right femoral neck, left femoral neck, and total body in all participants via dual-energy X-ray densitometry (DEXA) (enCORE™2008 Prodigy ID: 63283, Software V12.2, Copyright © 2008 GE Medical System Lunar, Madison, Wisconsin). Quality assurance was conducted once every morning before measuring each participant. Individual BMD values were expressed as Z-scores for group comparison. Normal bone density was defined as within 1 standard deviation (SD) of the young adult mean, osteopenic bone density was defined as between 1 and 2.5 SD below the young adult mean (T-score between -1 and -2.5) and osteoporotic bone density was defined as less than 2.5 SD below the young adult mean. Participants who exhibited bone density levels at the lower end of the young normal range (i.e., T-score less than 1 SD below the mean) were considered to be at increased risk of osteoporosis.

### 2.4. Laboratory Analysis

Liver and renal function and lipid profile tests were conducted in the biochemistry lab at Mubarak Hospital. The results were obtained and compiled for further analysis. Laboratory markers of bone turnover (procollagen type 1 amino-terminal propeptide [P1NP], C-terminal telopeptides [*β*-CTx or *β*-CrossLaps], osteocalcin, and intact parathyroid hormone [iPTH]) were determined by Cobas ECLIA (Roche Diagnostics GmbH, Mannheim, Germany). Serum total 25-hydroxyvitamin D (25-OH vitamin D) concentration was measured via competitive electrochemiluminescence (Roche Diagnostics, Mannheim, Germany) and serum calcitonin concentration was measured via ELISA (human Calcitonin ELISA Kit, REF EIA-3648, DRG International Inc., USA).

### 2.5. Statistical Analysis

The results are represented as the mean ± standard error of the mean (SEM). Statistical analysis was performed using SPSS Statistics for Windows, Version 17.0 (SPSS Inc., Chicago, IL, USA). Differences in anthropometric measurements, demographic information, and bone turnover markers across the three groups were assessed by Mann-Whitney tests and Pearson's chi-squared test. Differences in clothing style and BMD across the three groups were assessed by independent t-tests. Bone markers were analysed by one-way ANOVA. Spearman's correlations were conducted to assess the correlations between 25(OH)D and iPTH, calcitonin, BMD, and bone turnover markers and between bone turnover markers and iPTH and calcitonin within each group. In the final analysis, all three groups were combined to correlate changes between 25(OH)D and iPTH, calcitonin, BMD, and bone turnover markers; iPTH and bone turnover markers; and calcitonin and bone turnover markers.

## 3. Results

### 3.1. Participant Demographics

Biometric and demographic data were similar across all three groups (Tables 1 and 2). Family history of osteoporosis and supplement intake was not significantly different (Table 2). In addition, the duration of both indoor and outdoor sun exposure was similar across all three groups (Table 3). The majority of study participants reported spending 30 to 60 minutes outdoors during the morning and noon hours, times when UVB intensity is high (Table 3). All three groups also reported similar percentages of sun protection usage. Overall, there were no significant differences in sun exposure across the three groups, suggesting that any observed variability is due to clothing style. No significant differences were observed in any of the biochemical markers (Table 4).

### 3.2. BMD

No significant differences in BMD, Z-score, or bone mineral contents (BMC) were observed across the three groups (Table 5).

### 3.3. Bone Turnover Markers

Comparisons of bone turnover marker levels between the control and hejab groups and the control and veiled groups are reported in Table 6. The bone turnover marker osteocalcin was significantly higher in the hejab group compared to the control group (p<0.05). Both P1NP and *β*-CrossLaps were significantly higher (p<0.01 and p=0.001, respectively) in the veiled group compared to the control group.

### 3.4. 25(OH)D, iPTH, and Calcitonin Levels

**Figure 1:**
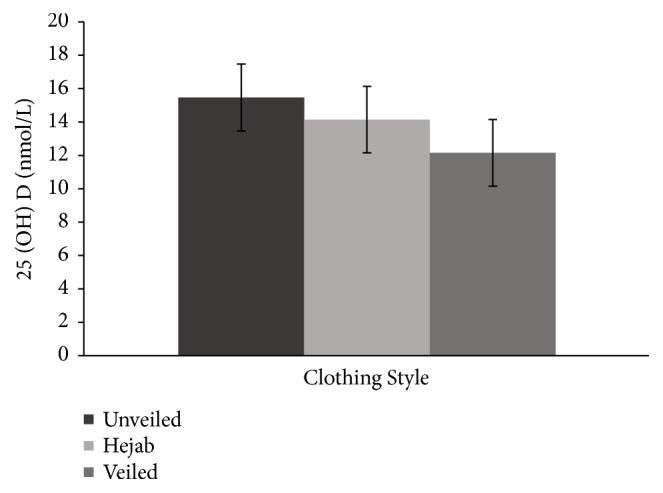
The mean (± standard error of the mean) 25-hydroxyvitamin D (25(OH)D) concentrations among the unveiled, hejab, and veiled groups. A decreasing trend was observed across the three groups.

**Figure 2:**
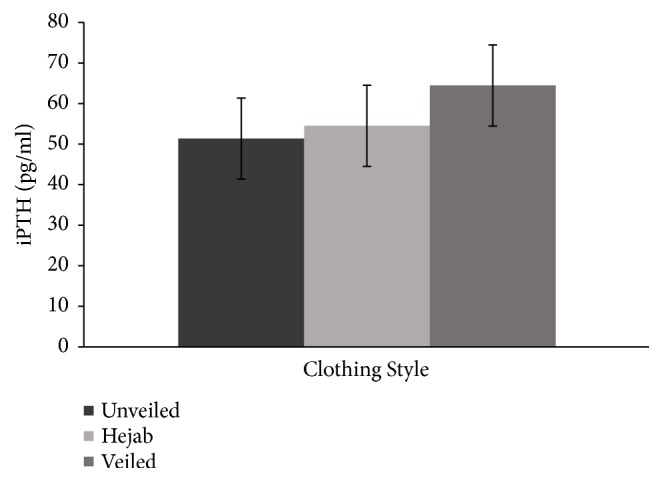
The mean (± standard error of the mean) intact parathyroid hormone (iPTH) concentrations among the unveiled, hejab, and veiled groups. An increasing trend was observed across the three groups.

**Figure 3:**
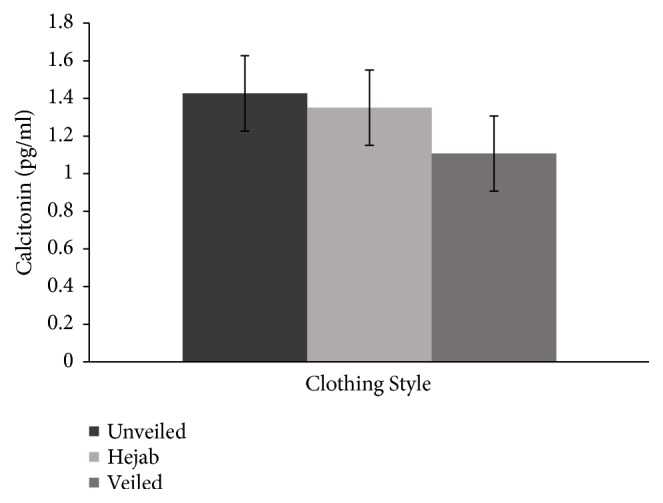
The mean (± standard error of the mean) calcitonin concentrations among the unveiled, hejab, and veiled groups. A decreasing trend was observed across the three groups.

The mean 25(OH)D level fell within the deficient range for all three groups. 25(OH)D was not significantly different across the three groups, but a linear decreasing trend was observed (Figure 1). Consistent with this finding, a linear increasing trend was observed in iPTH (Figure 2) and a linear decreasing trend was observed in calcitonin (Figure 3) across the three groups. iPTH levels were further classified into normal and abnormal ranges. Eight (26.7%) participants from the control group, 5 (17.2%) from the hejab group, and 12 (40%) from the veiled group had abnormally high iPTH levels (>65 pg/ml), indicating hyperparathyroidism. Normal iPTH and hyperparathyroidism were not significantly different across the three groups (Table 7).

## 4. Discussion

Vitamin D is known to impact bone health. Vitamin D deficiency therefore plays an important role in osteoporosis and other bone ailments [4]. Vitamin D levels are influenced by several factors, including age, ethnicity, exposure to UVB radiation, diet, body mass index (BMI), daily calcium intake, sunscreen use, and clothing style [18]

Vitamin D deficiency is frequently reported in individuals from Middle Eastern countries, including those in the Arabian Gulf, which are predominantly sunny year-round [12, 19–22]. In particular, vitamin D deficiency has been reported in Kuwaiti mothers and neonates [23] and adolescent Kuwaiti females [24]. Data published from Kuwait's first National Nutrition Survey described vitamin D status among Kuwaiti adults as “alarmingly low”, especially among Kuwaiti women [25].

The intent of the current study was to investigate whether clothing style plays a role vitamin D deficiency among Kuwaiti women. We focused on young, healthy, single Kuwaiti females in this study to control for other factors besides clothing style, such as menopausal status, that may cause discrepancies in vitamin D status. The body requires 10-15 minutes of exposure to solar UVB radiation between 10 am and 2 pm at least 2 to 3 times per week to synthesize adequate vitamin D levels [26]. All participants reported receiving at least 30 minutes of daily exposure to outdoor sunlight during the morning and noon hours, yet all groups exhibited vitamin D deficiency. We also asked about indoor sunlight exposure, as many women who wear the hejab or veil will remove these clothing items in their own homes, including in their gardens and yards, but did not detect significant differences in indoor sunlight exposure across the three groups. A decreasing trend in vitamin D levels was observed from the control to hejab to veiled groups, suggesting a role for clothing style in vitamin D deficiency. This finding agrees with other studies that have reported lower vitamin D levels among Arab/Muslim women in the Middle East [27, 28]. While these findings may in part be due to sun avoidance behaviours or low intake of vitamin D rich foods or supplements, it is notable that 95%-100% of Arab women cover their forearms, legs, and head when they go outdoors [19, 29]

Our findings are similar to findings that have been reported in other studies, including two studies in Saudi women [22, 30] and a study in women living in the United Arab Emirates (UAE) [31]. In this latter study, women of European ancestry who were living in the UAE exhibited higher levels of 25(OH)D than women native to the UAE and other women from non-Gulf Arab regions. Associations between clothing style and low vitamin D levels have also been reported in several other countries with adequate sunlight, including Turkey [4], Lebanon [19], Jordan [20], and Egypt [21]. Clothing style also has been associated with low vitamin D levels among Arab/Muslim females living in Western countries, including Denmark [32] and Sweden [33]. Together these findings indicate that clothing style related to religious and cultural practices may be a fundamental risk factor for vitamin D deficiency.

It has been postulated that UVB light is reflected via dust particles in polluted air, which is prominent during the summer months in most of the Gulf region. Although study participants asserted that they received more than 30 minutes of sunlight exposure each day, reflected UVB light due to air pollution and sun blocker usage may have limited their exposure. In addition, it is worth noting that, due to the high temperatures (sometimes exceeding 50°C) in Kuwait during the summer months, most individuals stay indoors during the day and use private cars as a means of transportation even for short distances and most of the cars are sun screened. These practices additionally limit sunlight exposure and may independently be associated with low vitamin D levels among Kuwaiti females.

### 4.1. Vitamin D and PTH

Bone remodelling is a continuous process by which old bone is replaced with new tissue to maintain skeletal shape, size, and quality. This process is regulated by a number of biochemical factors, including hormones and proteins secreted by both haemopoietin bone marrow cells and bone cells [34].

Vitamin D is involved in the regulation of calcium and phosphate levels in the blood by regulating their intestinal absorption, renal excretion, and bone calcium mobilization. Any drop in calcium levels stimulates PTH secretion, which in turn activates vitamin D synthesis. Vitamin D and PTH production enhances renal calcium reabsorption and calcium mobilization by bone resorption. However, elevated blood calcium levels promote a reduction in PTH secretion and consequently lead to a decrease in vitamin D synthesis and calcium mobilization. Increased blood calcium also stimulates parafollicular cells in the thyroid to secrete calcitonin, which inhibits calcium mobilization from the bone and stimulates calcium and phosphorous excretion. Thus, vitamin D, PTH, and calcitonin maintain calcium within normal levels [35, 36].

No significant difference in iPTH concentrations was observed across the groups in our study. A significant difference may have been observed if there had been a substantial difference in 25(OH)D concentrations across the groups and if the number of subjects was larger. Nevertheless, vitamin D status was negatively correlated with iPTH across all participants.

Twelve participants (40%) in the veiled group, 5 (17.2%) in the hejab group, and 8 (26.7%) in the control group exhibited elevated serum iPTH levels (>65 pg/ml), indicating the presence of secondary hyperparathyroidism. These findings are consistent with studies among women in Turkey, in which higher levels of PTH were observed in women who wore the veil and gloves compared to those who wore the hejab or Western-style clothing [4, 19]. Two studies have reported an inverse association between vitamin D and PTH levels, such that elevated PTH concentrations or high normal PTH concentrations are associated with 25(OH)D levels below 12-16 ng/ml [37–39]. The correlation between vitamin D and PTH hormone was further demonstrated in a cross-sectional study, in which data were collected between 2011 and 2013 for vitamin and PTH in Brazil, and the results obtained showed high prevalence of vitamin D deficiency and insufficiency, mainly among older people, yet younger people had been affected as well; the low vitamin D levels were found to be negatively correlated with PTH [40]. These findings are inconsistent, however, with the finding from another study that vitamin D levels were not significantly correlated with PTH levels [41], it has been proposed elsewhere that 30 to 125 nmol/L of 25(OH)D concentration is required to maintain normal PTH levels [42]. Furthermore, in a study of elderly subjects administered with vitamin D supplements of 400 IU/d, serum 25OHD increased from 19 to 25 ng/ml; nevertheless, no significant effect on serum PTH levels was observed [43]. Likewise, no changes in PTH levels were reported with even higher vitamin D supplements (4000 IU/d) given to a group of 55 year old subjects [44]. Thus, the proposed defining of vitamin D insufficiency as a serum vitamin D < than 30 ng/ml (75 nmol/liter), based on serum PTH suppression, is not supported by the literature review [43]. Consequently, it is difficult to conclude the type of relationship between vitamin D status and PTH; this is because of differences in methods used for assaying 25-hydroxy vitamin D and PTH, in addition, latitude, diet, race, age, and gender; all may have an impact on the correlation between vitamin D and serum PTH [45]. As mentioned earlier, the small number of participants in this study may have contributed to the lack of significant relationship between vitamin D and serum PTH.

In addition, comparative changes were observed in both serum calcitonin and 25(OH)D in all three groups. Serum calcitonin was lowest in the veiled group, which is consistent with the notion that calcitonin may play a role in stimulating vitamin D production under normocalcaemic conditions [46].

### 4.2. Bone Turnover Markers and BMD

Bone turnover and mineralization are affected by vitamin D status such that vitamin D deficiency may lead to low BMD [47]. Reduced BMD, which is also known as osteopenia, may lead to osteoporosis and an increased risk of fracture if left untreated [48, 49]. Vitamin D deficiency is prevalent among patients with osteoporosis [50]. Supplementation with vitamin D and calcium has been shown to reduce the risk of hip fractures among elderly women [51].

Prior studies are equivocal, however, regarding the direct relationship between vitamin D and BMD [49, 50, 52, 53]. In addition, high levels of bone turnover markers in the blood may indicate increased bone loss [54]. A cross-sectional study revealed that patients with fractures had higher bone turnover marker levels than individuals without fractures [55]. Another study reported that elevated levels of bone resorption markers can predict the likelihood of a future hip fracture even when bone density and mobility are unaffected [56].

In the present study, a significant difference in bone turnover marker levels was observed in both the hejab and veiled groups compared with the control group, suggesting that participants in both the hejab and veiled groups had higher bone turnover than participants in the control group. Several studies have reported that women have lower bone density than men [57]. It has been reported that women lose approximately 1% of their spinal bone density across the menstrual cycle and after menopause [58]. A cross-sectional investigation that included 635 healthy women of European descent demonstrated that those with the lowest bone mass had the highest levels of osteocalcin, NTX, CTX, and BALP [56]. Moreover, a 13-year longitudinal study in older women (mean age of 65 years) revealed that an increase in bone turnover markers, such as osteocalcin, was associated with a twofold increase in the risk of rapid bone loss [59]. Another study identified high levels of bone turnover markers as a risk factor for spinal fracture and osteoporosis [60], suggesting that routine bone turnover marker measurements may predict bone loss, future fractures, and the effectiveness of drugs. In contrast to these studies, our study was performed on young premenopausal female participants. Despite significant differences in bone turnover markers across the three groups of participants in our study, no participants exceeded the normal range for any bone marker.

Most prior studies have indicated that bone resorption markers are better predictors of bone loss than bone formation markers [59, 61]. A study in elderly women with low bone density and with or without spinal fractures had high levels of *β*-CrossLaps and osteocalcin. Another study showed a significant correlation between spinal fractures and both of these bone turnover markers. *β*-CrossLaps is a bone resorption marker, whereas osteocalcin is a bone formation marker; thus, both marker types may be predictors of bone loss [62]. In our study, osteocalcin was significantly higher in participants wearing the hejab compared with the control group, whereas P1NP, another bone formation marker, and *β*-CrossLaps were higher in veiled participants. These participants also exhibited higher rates of bone loss than participants in the control group. Vitamin D together with vitamin D receptor (VDR) directly activates human osteocalcin (hOC) gene expression through a vitamin D-responsive element (VDRE) located in the promoter of the hOC gene [63]. Therefore it is expected to notice a significant drop in osteocalcin levels among the veiled group as they have the lowest vitamin D levels; however, all groups expressed vitamin D deficiency with no significant differences between them. Thus using osteocalcin as a bone marker is not advisable in case of vitamin D deficiency.

Several studies have shown that biochemical bone turnover markers increase at puberty and then return to normal levels as bone formation and resorption reach equilibrium [56, 64]. These bone markers then become unbalanced again after the age of 40. Thus, high bone turnover markers are not always predictors of bone loss. Elevation of these markers may occur as a result of growth or during treatment with PTH [59]. In the present study, our subjects were young but postpubertal (20-35 years old).

DEXA is another technique used to assess bone strength by determining BMD. Whereas bone markers provide an overall picture of bone turnover, BMD measures specific areas of the skeletal system. Although there were no significant differences observed in overall BMD across the three groups in our study, mean femoral neck BMD was lower in the hejab and veiled groups compared with the control group. Nevertheless, bone turnover markers may predict changes in bone before any significant change can be detected in BMD. For example, in a study by Bauer and colleagues, patients with osteoporosis were treated for 1 year with alendronate, a bisphosphonate. Significant changes in biochemical bone markers were observed 3 to 6 months after the start of treatment, whereas significant changes in BMD were not observed for 18 months [65]. A similar observation has been reported by Melton et al. [66]. In addition, the negative correlation between BMD and bone markers becomes stronger as age increases [56]. Thus, in our study, participants may have been too young to exhibit significant changes in bone density. Bone turnover markers therefore provide an instantaneous measure of bone turnover that can be obtained more rapidly than BMD [67].

Because study participation involved providing blood samples and exposure to radiation for bone mineral densitometry, a limited sample size of 30 volunteers from each group was analysed. Therefore, the results of our study should be interpreted with caution, and larger sample sizes are required in future studies to generate results with adequate statistical power to unravel the effect of clothing style on vitamin D and bone turnover.

## 5. Conclusion

The present study is one of the few studies in the existing literature to evaluate both bone markers and BMD in relation to clothing style. The findings from this study revealed a positive association between high bone turnover markers and traditional or cultural clothing style. These high bone turnover marker levels may be predictors of increased risk of low bone density, which in turn may lead to a higher risk of osteoporosis.

Most food products in Kuwait and many other countries in the Middle East are not fortified with vitamin D. Residents of these countries therefore must rely on vitamin D_3_ synthesis via sunlight exposure. Our cohort revealed low vitamin D levels, low calcitonin levels, and high iPTH levels in all participants, even those who wore Western-style clothing. Although the majority of our participants had vitamin D deficiency, participants who wore the hejab or were veiled had lower vitamin D levels than those who wore Western-style clothing. This study therefore suggests that clothing style may contribute to vitamin D deficiency. Clothing style, however, is not considered the primary causative factor of low vitamin D levels among the Kuwaiti population. Intense heat during the summer months limits direct outdoor exposure to sunlight. Thus, the primary causes of vitamin D deficiency vary worldwide and may include factors such as ethnicity, lifestyle, living conditions, dietary status, and physical movement.

## Figures and Tables

**Table 1:** Biometric data.

	Unveiled (n=30)	Hejab (n=30)	Veiled (n=30)
Age	21.93 ± 0.358	24.1 ± 0.606	22.15 ± 0.395
Height (m)	1.6 ± 0.011	1.6 ± 0.013	1.59 ± 0.012
Weight (kg)	62.64 ± 2.57	65.35 ± 3.75	67.76 ± 2.71
BMI (kg/m^2^)	24.46 ± 1.11	25.15 ± 1.27	26.62 ± 1.01

All variables are expressed as the mean ± standard error of the mean (SEM). Pearson's chi-squared test was used for comparisons between groups; no significant differences were detected. BMI, body mass index.

**Table 2:** Demographic information.

	Unveiled (n=30)	Hejab (n=30)	Veiled (n=30)
Exercise	11(36.7%)	12 (40%)	15 (50%)
Smoking	3 (10%)	0 (0%)	0 (0%)
Milk Consumption	21 (70%)	25 (83.3%)	22 (73.3%)
History of Osteoporosis	8 (26.7%)	7 (23.3%)	2 (6.7%)
Supplement Intake	5 (16.7%)	1 (3.3%)	5 (16.7%)

All variables are expressed as the number of participants per group (percentage). Pearson's chi-squared test was used for comparisons between groups; no significant differences were detected.

**Table 3:** Indoor and outdoor sun exposure.

		Unveiled (n=30)	Hejab (n=30)	Veiled (n=30)
		Frequency (%)		
Duration of Indoor Sun Exposure (minutes)	0	20 (66.7%)	16 (53.3%)	16 (53.3%)
	<30	10 (33.3%)	8 (27%)	9 (30%)
	30-60		3 (10%)	3 (10%)
	60-120		2 (7%)	1 (3%)
	>120		1 (3%)	1 (3%)

Duration of Outdoor Sun Exposure (minutes)	0	1 (3.3%)	1 (3.3%)	0 (0%)
	<30	13 (43.3%)	12 (40%)	13 (43.3%)
	30-60	14 (46.7%)	13 (43.3%)	13 (43.3%)
	60-120	2 (6.7%)	4 (13.3%)	1 (3.3%)
	>120	0 (0%)	0 (0%)	3 (10%)

Time of Exposure	Morning	10 (33.3%)	9 (30%)	18 (60%)
	Noon	16 (53.3%)	16 (53%)	11 (37%)
	Afternoon		4 (13.3%)	1 (3.3%)

Use of Sun Protection	No	25 (83.3%)	29 (96.7%)	25 (83.3%)
	Yes	5 (16.7%)	1 (3.3%)	5 (16.7)

All variables are expressed as the number of participants per group (percentage). Pearson's chi-squared test was used for comparisons between groups; no significant differences were detected.

**Table 4:** Fasting biochemical variables.

	Unveiled (n=30)	Hejab (n=30)	Veiled (n=30)	Reference Range
GLUC	3.8 ± 0.2	3.6 ± 0.1	3.9 ± 0.2	3.9-6.1 mmol/L
*Kidney Profile*				

BUN	3.21 ± 0.17	3.25 ± 0.2	2.81 ± 0.15	2.5-6.6 mmol/L
CREA	41.2 ± 2.62	41.8 ± 1.85	38.7 ± 2.32	60-120 *μ*mol/L
URIC	227 ± 9.23	235 ± 9.17	215 ± 10.9	150-400 *μ*mol/L
Na	131.3 ± 3.75	128.5 ± 2.59	129.3 ± 2.7	135-148 mmol/L
K	3.75 ± 0.11	3.84 ± 0.11	3.64 ± 0.1	3.5-5.3 mmol/L
*Liver Profile*				

ALB	33.5 ± 1.74	31.5 ± 1.11	34.1 ± 1.48	35-47 g/L
ALP	34.1 ± 2.97	32.6 ± 1.91	36.1 ± 3.1	26-88 IU/L
ALT	11 ± 1.08	8.94 ± 0.53	9.25 ± 0.94	10-60 IU/L
AST	15 ± 1	13 ± 1.1	14 ± 1	10-42 IU/L
TP	58 ± 3.03	59.6 ± 4.51	61.1 ± 2.3	63-80 g/L
*Lipid Profile*				

CHOL	3.19 ± 0.23	3.39 ± 0.14	3.3 ± 0.19	3-5.2 mmol/L
TG	0.67 ± 0.06	0.83 ± 0.08	0.66 ± 0.05	0-2 mmol/L
HDL	1.01 ± 0.08	1.13 ± 0.04	3.29 ± 2.06	0.9-2.5 mmol/L
*Others*				

Corrected Ca	2.23 ± 0.035	2.25 ± 0.199	2.22 ± 0.035	2.2-2.6 mmol/L
PO_4_	0.89 ± 0.06	0.92 ± 0.03	1.04 ± 0.06	0.8-1.4 mmol/L
IRON	16.3 ± 1.57	16.6 ± 1.36	14.4 ± 1.8	11-31 *μ*mol/L
TRFN	2.7 ± 0.08	3.5 ± 0.1	3.3 ± 0.16	2.1-3.6 g/L

All variables are expressed as the mean ± standard error of the mean (SEM). GLUC, glucose; BUN, blood urea nitrogen; CREA, creatinine; URIC, uric acid; Na, sodium; K, potassium; ALB, albumin; ALP, alkaline phosphatase; ALT, alanine aminotransferase; AST, aspartate aminotransferase; TP, total protein; CHOL, cholesterol; TG, triglycerides; HDL, high-density lipoprotein; Ca, calcium; PO_4_, phosphate; TRFN, transferrin.

**Table 5:** Bone mineral density comparisons.

	Unveiled (n=30)	Hejab (n=30)	Veiled (n=30)
Left Femoral Neck			
BMD (g/cm^2^)	0.99 ± 0.02	0.97 ± 0.028	0.98 ± 0.022
Z-score	-0.43 ± 0.16	-0.62 ± 0.22	-0.58 ± 0.17
Right Femoral Neck			
BMD (g/cm^2^)	0.99 ± 0.02	0.96 ± 0.03	0.98 ± 0.019
Z-score	-0.41 ± 0.16	-0.66 ± 0.195	-0.61 ± 0.15
Lumbar Spine (L2-L4)			
BMD (g/cm^2^)	1.19 ± 0.02	1.17 ± 0.025	1.24 ± 0.02
BMC (g)	45.8 ± 1.39	44.3 ± 1.45	49.4 ± 1.53
Z-score	0.028 ± 0.18	-0.24 ± 0.21	0.27 ± 0.16
Total Body			
BMD (g/cm^2^)	1.13 ± 0.014	1.12 ± 0.018	1.16 ± 0.014
BMC (g)	2340.3 ± 59.81	2298.5 ± 54.6	2384.7 ± 64.32
Z-score	0.2 ± 0.13	-0.02 ± 0.17	0.25 ± 0.12

All variables are expressed as the mean ± standard error of the mean (SEM). Independent t-tests were conducted for comparisons between groups; no significant differences were detected. BMD, bone mineral density; BMC, bone mineral contents.

**Table 6:** Bone turnover marker comparisons between the unveiled and hejab groups.

Bone Marker	Unveiled	Veiled	Hejab	Reference Range
	(n=30)	(n=30)	(n=30)	ng/ml
P1NP	55.13 ± 3.69	73.8 ± 4.52*∗∗*	62.12 ± 4.6	19-100
Osteocalcin	23.22 ± 1.39	26.63 ± 1.46	31.23 ± 2.52*∗*	0.5-300
*β*-CrossLaps	0.39 ± 0.03	0.54 ± 0.03*∗∗∗*	0.41 ± 0.03	0.112-1.081

All variables are expressed as the mean ± standard error of the mean (SEM). The Mann-Whitney test was conducted for comparisons between groups. P1NP and *β*-CrossLaps were significantly higher in the veiled group compared to unveiled one; *∗∗*p<0.01 and *∗∗∗*p=0.001, respectively.

Osteocalcin was significantly higher in the hejab group compared to unveiled group; *∗*p<0.05.

**Table 7:** Intact parathyroid hormone levels across groups.

iPTH		Unveiled	Hejab	Veiled	*p *Value
		(n=30)	(n=30)	(n=30)	
Normal (≤65 pg/ml)	Frequency	22	24	18	NS
	%	34.40%	37.50%	28.10%	
High (>65 pg/ml)	Frequency	8	5	12	NS
	%	32%	20%	48%	

All variables are expressed as the number of participants per group (percentage). Pearson's chi-squared test was conducted for comparisons between groups. iPTH, intact parathyroid hormone; NS, not significant.

## Data Availability

The authors confirm that the data supporting the finding of this study are available with the article as tables and figures.
